# Antibacterial and anti-biofilm properties of carvacrol alone and in combination with cefixime against *Escherichia coli*

**DOI:** 10.1186/s12866-023-02797-x

**Published:** 2023-03-03

**Authors:** Sepideh Asadi, Bahar Nayeri-Fasaei, Taghi Zahraei-Salehi, Ramak Yahya-Rayat, Nemat Shams, Aram Sharifi

**Affiliations:** 1 Department of Microbiology and Immunology, University of Tehran, Faculty of Veterinary Medicine, Tehran, Iran; 2 Department of Pathobiology, Lorestan University, Faculty of Veterinary Medicine, Tehran, Iran; 3 Department of Animal Science, University of Kurdistan, Faculty of Agriculture, Sanandaj, Iran

**Keywords:** *Escherichia coli*, Anti-quorum sensing, Anti-biofilm, Carvacrol, Cefixime

## Abstract

**Background:**

Plant-derived compounds can be used as antimicrobial agents in medicines and as food preservatives. These compounds can be applied along with other antimicrobial agents to strengthen the effect and/or reduce the required treatment dose.

**Results:**

In the present study, the antibacterial, anti-biofilm and quorum sensing inhibitory activity of carvacrol alone and in combination with the antibiotic cefixime against *Escherichia coli* was investigated. The MIC and MBC values for carvacrol were 250 μg/mL. In the checkerboard test, carvacrol showed a synergistic interaction with cefixime against *E. coli* (FIC index = 0.5). Carvacrol and cefixime significantly inhibited biofilm formation at MIC/2 (125 and 62.5 μg/mL), MIC/4 (62.5 and 31.25 μg/mL) and MIC/8 (31.25 and 15.625 μg/mL) for carvacrol and cefixime, respectively. The antibacterial and anti-biofilm potential effect of carvacrol confirmed by the scanning electron microscopy. Real-time quantitative reverse transcription PCR revealed significant down-regulation of the *luxS* and *pfs* genes following treatment with a MIC/2 (125 μg/mL) concentration of carvacrol alone and of only *pfs* gene following treatment with MIC/2 of carvacrol in combination with MIC/2 of cefixime (*p* < 0.05).

**Conclusions:**

Because of the significant antibacterial and anti-biofilm activity of carvacrol, the present study examines this agent as an antibacterial drug of natural origin. The results indicate that in this study the best antibacterial and anti-biofilm properties are for the combined use of cefixime and carvacrol.

## Background

A microbial biofilm is a community of microbial cells that are attached to a living or non-living surface, and are enclosed in an external matrix. Biofilm cells use different mechanisms to resist antimicrobial compounds [1–4]. Their resistance sometimes increases to up to 1000 times that of free-phase or planktonic cells. Biofilms in the body are also resistant to the defense of the immune system [5]. In food processing systems such as factories, biofilms are hardly removed by conventional disinfectants and can act as a source of contamination. This issue has caused the study of anti-biofilm compounds to expand and attract the attention of researchers [1, 6–8]. Gram-positive bacteria such as *Staphylococcus* spp. and *Enterococcus* spp. and gram-negative bacteria such as *Escherichia coli*, *Pseudomonas aeruginosa* and *Acinetobacter* have the ability to form biofilms [9–11]. Scientists have suggested different mechanisms and agents for the removal of such bacterial biofilm. These include long-term antibiotic therapy, photodynamic therapy, antifouling agents, biofilm dissolving substances and quorum sensing (QS) inhibitors [12]. The intercellular communication, or QS system, controls bacterial mechanisms such as bacterial growth and multiplication, toxin production, and biofilm formation [1, 13]. Because bacterial QS controls biofilm formation, substances or mechanisms that disrupt this system are known as biofilm inhibitors. As QS inhibitors do not affect bacterial growth, they do not lead to microbial resistance or the emergence of multi-drug-resistant strains [1, 14]. It has been shown that plant-derived compounds can affect bacterial biofilms in different ways such as those affecting the bacterial QS system. These compounds not only inhibit bacterial biofilm, but also affect virulence-related factors such as bacterial efflux pumps and toxin production [13, 15].

*Escherichia coli* is one of the most common microorganism of the intestinal normal flora. *E. coli* able to survive in water for 4–12 weeks, and it appears as an indicator to provide the accurate bacterial contamination of fecal matter in foods and drinking water [15, 16].

The most important pathotype of *E. coli* includes Enteroaggregation *E.coli* (EAEC), Enterohemorrhagic *E. coli*(EHEC), Enterinvasive *E. coli* (EIEC), Enteropathogenic *E. coli* (EPEC), Enterotoxigenic *E. coli* (ETEC) and Diffusely Adherence *E.coli* (DAEC). EHEC is known as the most causative agent of zoonotic disease between humans and animals. This strain could be transmitted to humans by consuming contaminated foods and leads to bloody diarrhea and high mortality [16, 17].

Previous studies have shown that *E. coli* has the ability to produce a strong biofilm that can both increases its pathogenicity and make it resistant to antimicrobial drugs [18–21]. During the biofilm formation, autoinducer 2 (AI-2) signals derived from furanone increase its adaptation to different environmental conditions, its adhesion ability, and promote biofilm formation, which will result in a high level of pathogenicity [22, 23]. The *luxS* and *pfs* genes are considered to be the most important ones involved in the production of acyl-homoserine lactone. In addition to the production of AI-2 signals, the *luxS* gene is involved in the production of AI-3 signals in EHEC that promote intestinal colonization and ulcers [22, 24, 25].

One of the most important approaches for the treatment of infectious diseases is the use of different antibiotics. However, the overuse of antibiotics in human and veterinary medicine and in industry has led to the emergence of drug-resistant strains. This has become a growing public health concern worldwide [26–28]. Cefixime is a third-generation cephalosporin that can be used as a second line of treatment in Gram-negative bacteria after resistance to aminopenicillins, cefaclor and sulfonamides [29]. Based on reports, greater than 90% of *E. coli* isolates was susceptible to cefixime. In addition, cefixime is effective drug for uncomplicated urinary tract infections (UTI) caused by *E. coli *[30].

Carvacrol, or cymophenol (2-methyl-5-propan-2-ylphenol), is a monoterpene phenolic compound obtained from the essential oils of members of the Labiatae family, including *Origanum*, *Satureja*, *Thymbra*, *Thymus* and *Corydothymus *[31]. The biological properties of carvacrol include its anti-oxidant, anti-inflammatory, anti-cancer, anti-pyretic, and analgesic properties [32]. The antibacterial and anti-biofilm properties of carvacrol have been reported in previous studies [32–34]. Carvacrol has also been reported to be safe and exert minimal toxicity toward human cells [32]. Although there have been many reports on the antimicrobial activities of carvacrol [32, 34], studies on the synergistic antimicrobial effects of this agent with antibiotics have been limited [35]. The present study was aimed at determining the antibacterial, anti-biofilm and quorum sensing inhibitory activity of carvacrol against *E. coli* as well as the synergistic effect of this compound with the antibiotic cefixime.

## Results

### Antibacterial potential of carvacrol and cefixime

The results of the disk diffusion test showed that *E. coli* was sensitive to cefixime (5 μg/ disk) (mean growth inhibition zone of 23 ± 4 mm). The mean growth inhibition zones for carvacrol at concentrations of 1000, 500, and 250 μg/mL were 38 ± 5, 30 ± 4, and 18 ± 4 mm, respectively. The results for MIC and MBC are given in Table 1 and showed growth inhibitory potential and bactericidal activity against *E. coli* for both carvacrol and cefixime.

**Table 1:** Antibacterial activity of carvacrol and cefixime against *E. coli*

Bacteria	Test	Carvacrol	Cefixime
*E. coli*	MIC (μg/mL)	250	125
	MBC (μg/mL)	250	125

*MIC* minimum inhibitory concentration, *MBC* minimum bactericidal concentration

### Interaction between carvacrol and cefixime

The interaction between carvacrol and cefixime was measured using the checkerboard test. The FIC_I_ of the combination of carvacrol and cefixime was 0.5 (FIC carvacrol = 0.25 and FIC cefixime = 0.25). The interaction of carvacrol with cefixime showed significant synergistic effects (*p* < 0.05). This means that this combination significantly reduced the MIC of carvacrol and cefixime against *E. coli*.

### Anti-biofilm potential of carvacrol and cefixime

**Fig. 1:**
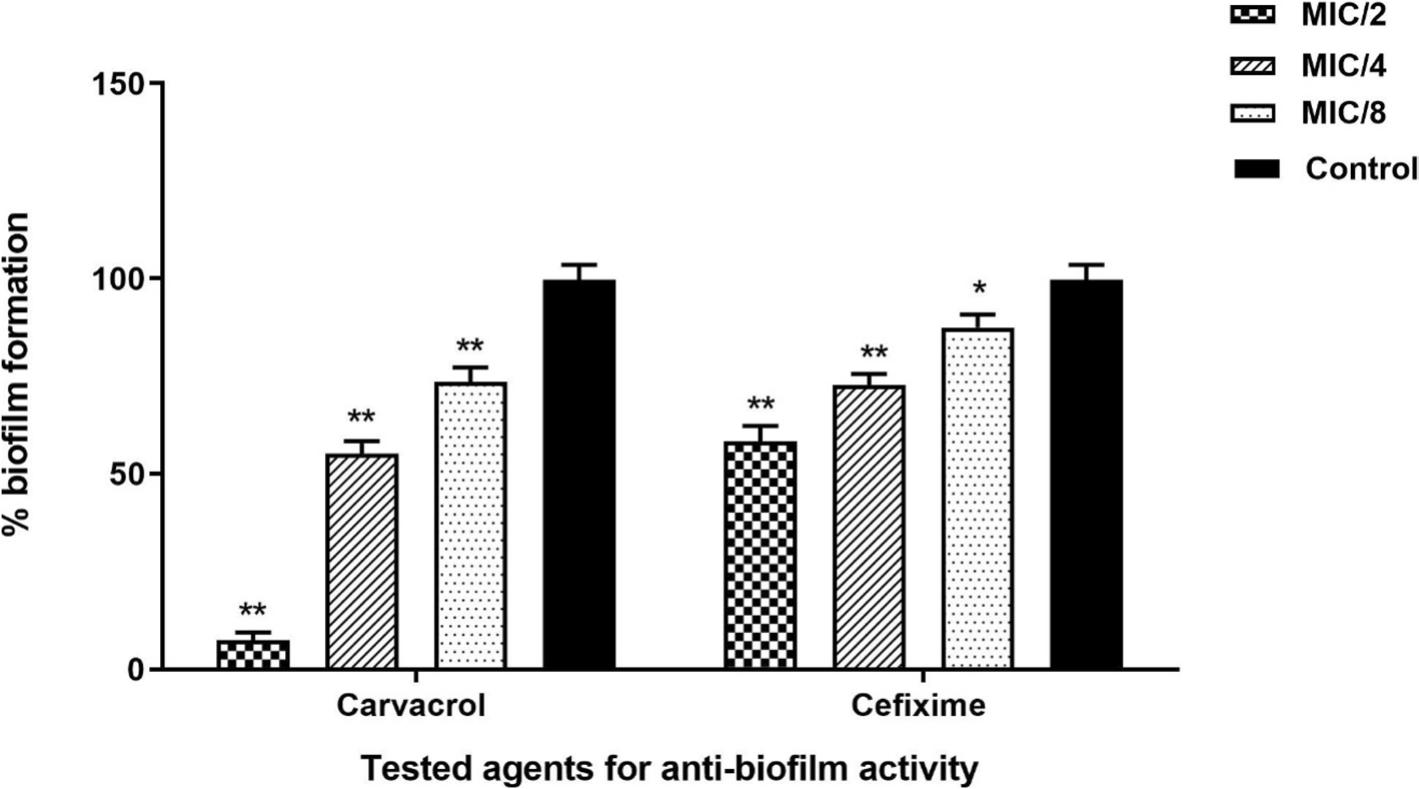
Anti-biofilm potential effect of carvacrol and cefixime against *E. coli*. The error bars represent the SD of three replicates (*p < 0.05 and ** *p* < 0.001)

Based on the results obtained on biofilm formation through the CV staining method, both carvacrol and cefixime resulted to have anti-biofilm properties effect that were dose dependent. Most of the anti-biofilm properties were related to MIC/2 followed by MIC/4 and MIC/8 concentrations. However, all three concentrations (MIC/2, MIC/4 and MIC/8) significantly inhibited biofilm formation by *E. coli* (*p* < 0.05). It should be noted that the anti-biofilm properties of carvacrol were stronger than of cefixim (Fig. 1).

### SEM of bacterial cell structure and biofilm

**Fig. 2:**
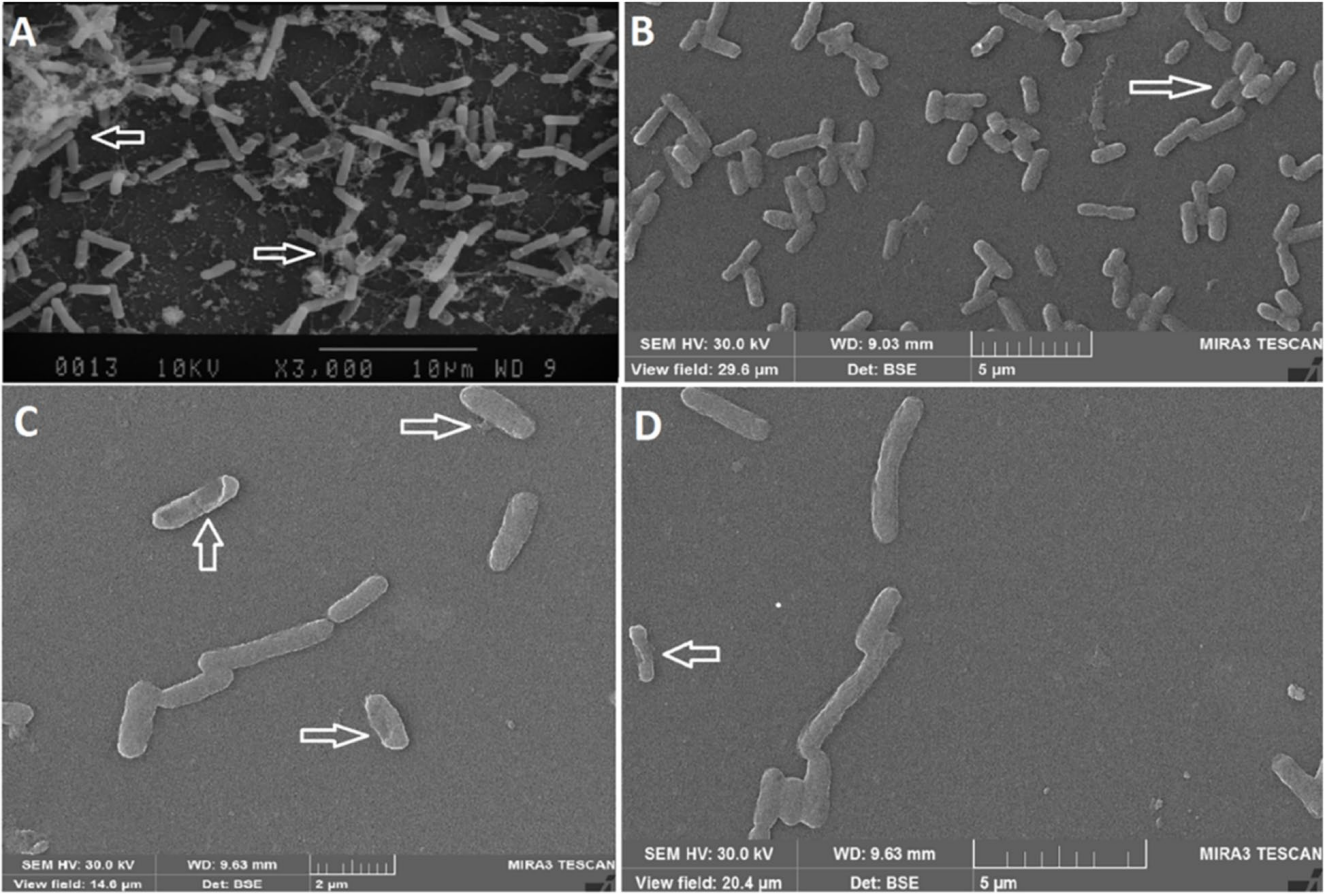
SEM images of bacterial cells: **A** control (without carvacrol and cefixime) showing strong cell condensation, biofilm formation and extracellular exopolysaccharide in the biofilm with healthy bacterial cells and intact wall; **B** culture containing MIC/2 cefixime showing much reduced, but visible, biofilm (white arrow); **C** culture containing MIC/2 carvacrol showing absence of biofilm and destruction of wall; **D** culture containing MIC/2 cefixime + MIC/2 carvacrol showing destruction and removal of biofilm (white arrow)

The bacterial cell structure and architecture of the biofilms formed in presence of with the MIC/2 concentration of carvacrol or cefixime and carvacrol/cefixime combinations were analyzed by SEM. Figure 2 shows a significant decrease in the number of adherent bacterial cells as well as the size of aggregates between the control and carvacrol/cefixime treated samples. In addition, the cell structure in the sample treated with carvacrol can be observed have changed and the cell integrity was being lost (Fig. 2).

### Quantification of QS gene expression

**Fig. 3:**
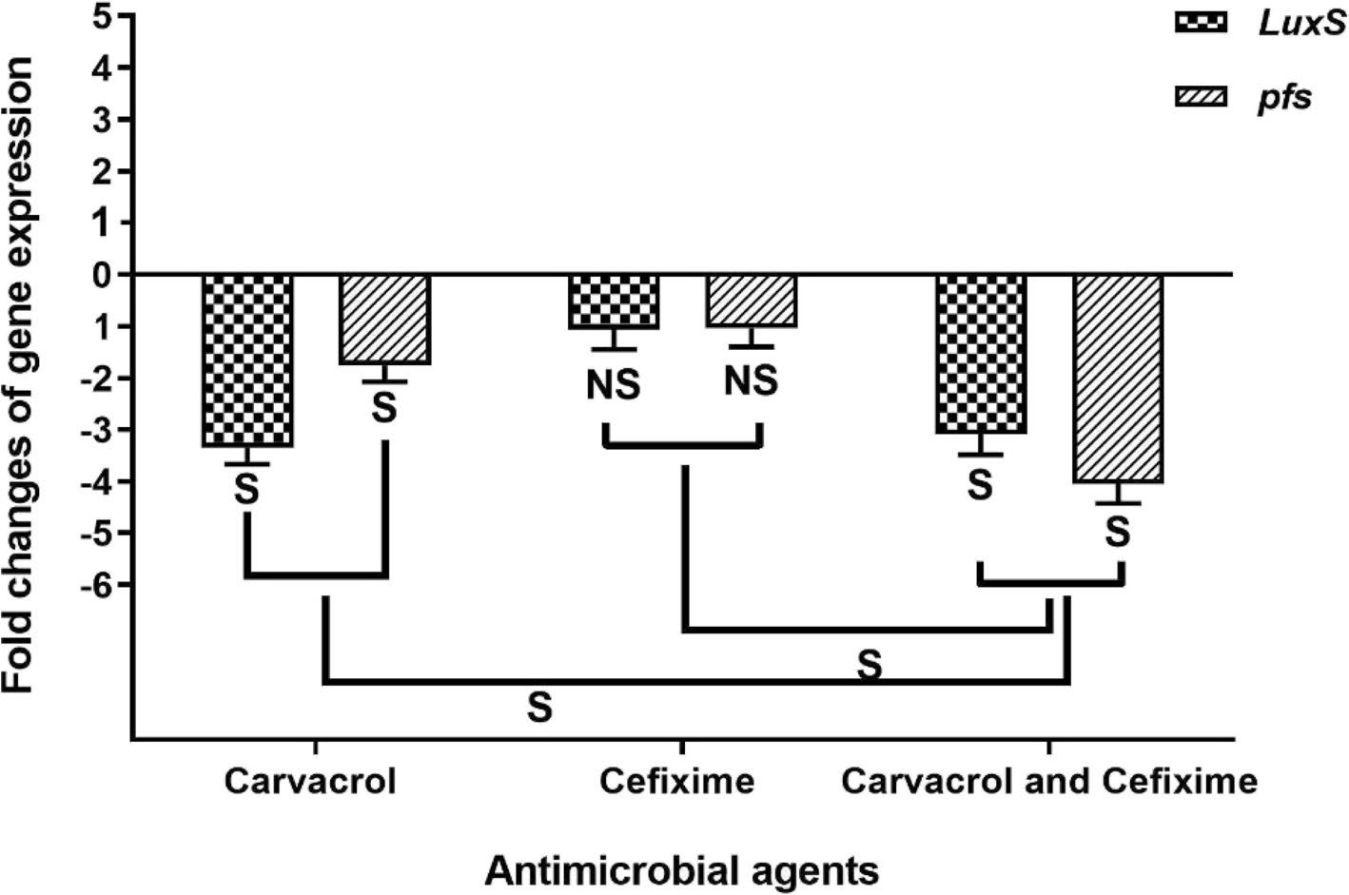
Effect of MIC/2 concentration of carvacrol and cefixime on *luxS* and *pfs* expression. the expression level of the two genes are reported as levels of induction (n-fold) in treated cells relative to that of untreated bacteria. S: significant, NS: non-significant (*p* > 0.05)

The effects of the MIC/2 concentration of carvacrol and cefixime alone and in combination on the expression of two QS-related genes were measured in the biofilm phase of *E. coli*. The MIC/2 concentration of carvacrol caused a significant down regulation of both *luxS* (*p* < 0.022) and *pfs* (*p* < 0.037). The effect of cefixime on the expression of *luxS* and *pfs* was not significant (*p* > 0.05; Fig. 3). It should be noted that in the combined use of carvacrol and cefixime, the expression of the only *pfs* gene significantly decreased compared to when carvacrol was used alone.

## Discussion

Since ancient times, medicinal plants, and their compounds such as carvacrol have been used in traditional medicine. Their different biological properties and, especially, their antimicrobial properties, now can be used to preserve the quality of food and increase its shelf life [36, 37]. Additionally, due to the increase in antibiotic resistance, there is a vital need for new drugs with multiple mechanisms of action [38, 39]. Carvacrol is a monoterpenoid phenolic derivative found in some herbal plants, including oregano and thyme. Because of the different biological properties of carvacrol, many studies have been performed on this compound to evaluate its usability in medicine [32, 35, 40]. The present study investigated the antimicrobial, anti-biofilm and anti-QS activities of carvacrol against the important foodborne pathogen *E. coli*. Both the MIC and MBC values of carvacrol against *E. coli* were 250 μg/mL. The SEM images showed the antimicrobial properties of cefixime and carvacrol and their combination. The results were consistent with what has been previously reported for the antibacterial properties of carvacrol against Group A Streptococci (MIC and MBC of 125 and 250 μg/mL, respectively) [35]. In a similar study, the antibacterial activity of carvacrol was reported against *Bacillus subtilis*, *Enterobacter cloacae*, *E. coli* O157:H7, *Micrococcus flavus*, *Proteus mirabilis*, *Pseudomonas aeruginosa*, *Salmonella enteritidis*, *Staphylococcus epidermidis*, *Salmonella typhimurium*, and *Staphylococcus aureus* at MIC values of 0.02–0.5 μg/mL and MBC values of 0.125–1.0 μg/mL [41]. Ben Arfa et al. (2006) reported on the antibacterial properties of two types of carvacrol-derived molecules (carvacrol methyl ether and carvacrol acetate) against the following Gram negative bacteria, Gram-positive bacteria and yeast: *E. coli, Pseudomonas fluorescens*, *S. aureus*, *B.*, *Lactobacillus plantarum*, *Saccharomyces cerevisiae *[42]. The results of these studies along with those of the present study show that carvacrol and its derivatives have antimicrobial properties against various types of bacteria and fungi. Most of the studies on the mechanism of action of this compound show that the main target of carvacrol is the cytoplasmic membrane of the bacteria [35, 42]. Carvacrol can permeate the structure of the cell membrane and cause disintegration of the outer membrane of microbial cells [43]. This membrane damage affects the pH homeostasis and equilibrium of the inorganic ions, leading to the induction of antibacterial activity of this substance [44]. In addition, as the cytoplasmic membrane plays a vital role in prokaryotic cells viability, this membrane destruction can cause cell death [44]. Recent studies have shown that bacterial isolates, including *E. coli*, are highly resistant to common antibiotics such as cefixime [45, 46]. A high rate of resistance was reported for cefixime (57.9%) on *E.coli* isolated. from children at at Shahid Sadoughi Hospital in Yazd. In another study in Iran performed on *E. coli* isolated from urinary tract infections, the resistance to cefixime was 50% [47]. The results of these and similar studies, it can be concluded that *E. coli* strains in Iran are highly resistant to cefixime. As a result, it is crucial to find a compound to restore sensitivity to cefixime. The present study also investigated the synergistic effect of carvacrol and cefixime against *E. coli* which, to our knowledge, has never been investigated. The results shown that carvacrol in combination with cefixime possessed a synergistic action against *E. coli*. Previous studies have reported on synergistic antibacterial activity of various plant-derived compounds in the presence of conventional antibiotics [48, 49]. For example, synergistic interactions of cinnamic acid, cinnamaldehyde, eugenol and thymol with several conventional antibiotics have been reported [50]. The combination of these phytochemicals with antibiotics have led to the reduction of the minimum effective dose of the antibiotics required for treatment, which can reduce the adverse effects of these drugs [51]. In addition to reducing the minimum effective dose of antibiotics, plant-derived compounds can reflect the modification or blocking of resistance mechanisms such that the bacterium becomes sensitive to an antibiotic or the antibiotic is active when used at lower concentrations [49]. Several studies have examined the synergistic effects of carvacrol and antibiotics. Similar to the present study, one study showed that carvacrol significantly reduced the MIC of erythromycin against erythromycin-resistant Group A streptococci [35]. The synergistic effects of carvacrol have been reported in combination with antibiotics as well as with other the biological compounds of thymol [44], eugenol [52], nisin [53] and organic acids such as acetic acid, lactic acid, and citric acid [54]. Pol and Smid investigated the combined interaction of carvacrol and nisin against *Listeria monocytogenes* and *Bacillus cereus*. These researchers reported that carvacrol was able to increase the antibacterial activity of nisin by increasing the number or size of the pores in the cytoplasmic membrane created by nisin and lengthened the lifetime of the pores or both of them [55]. The current study reports on the synergistic properties of carvacrol and the antibiotic cefixime; but further studies will be required to determine its mechanism of action. In the present study, the anti-biofilm and QS inhibitory activity of carvacrol and cefixime were investigated against *E. coli*. The formation of biofilm by *E. coli* helps to determine its effective pathogenesis. On the other hand, the creation of biofilm with various mechanisms increases drug resistance against different antibiotics. Accordingly, inhibition of biofilm formation at sub-lethality concentrations can be very important. In this concentration it is unlikely to develop multidrug resistant pathogens since it does not impose any selection pressure [13, 56–58]. Figure 1 shows that, at MIC/2, MIC/4 and MIC/8, both carvacrol and cefixime significantly reduced the biofilm formation by *E. coli* (*p* < 0.05). The anti-biofilm properties of carvacrol were found to be significantly stronger than cefixime (*p* < 0.05). The anti-biofilm properties of various compounds are exerted in different ways. These include membrane disruption, substrate deprivation, binding to adhesin complex, proteins, interaction with DNA and blocking viral fusion [6]. Both natural and synthetic agents also have been introduced for their anti-biofilm properties, such as metal nanoparticles [59], plant extract and essential oils [1, 60], antimicrobial peptides [61], antibiotic [62], and QS inhibitory agents [1]. Several studies have examined the anti-biofilm properties of this compound against pathogenic bacteria [63–65]. A similar study reported that carvacrol can inhibit the biofilm formation of *Salmonella enterica* serovar Typhimurium at MIC and MIC/2 concentrations [34]. As stated, one of the strategies to control biofilm formation is the use of a compound that inhibits or destroys the bacterial QS system [1, 66]. This has prompted many studies on QS inhibitors, such as plant derivatives, and studies have shown that plant derivatives can well disrupt this system. Because carvacrol has a destructive effect on cell membranes, it has been hypothesized that it has anti-biofilm properties [6, 67]. Previous studies demonstrated that SEM images are suitable tools for investigating and studying bacterial biofilm and cell structure [1, 14]. The present study presented SEM images of the reduction of bacterial biofilm and extracellular exopolysaccharide production, as well as the bacterial destruction caused by carvacrol and at a lesser extent by cefixime (Fig. 2). It has been shown that carvacrol in both conditions (alone and in combination with the cefixime) significantly reduced the expression of QS-related genes of *E. coli*. The most decrease in *pfs* gene expression occurred when carvacrol was used together with cefixime (Fig. 3). This reduction in expression reduced the production of autoinducer molecules and disrupted the biofilm formation process. Considering that the QS system is involved in bacterial cell growth, proliferation, motility, toxin production and antibiotic resistance, it suggested that this decrease in expression may inhibit these cellular mechanisms [13]. As anti-virulence agents do not impose life or death selective pressures in bacteria, using them to control infectious diseases is considered and suggested by researchers [14, 15].

## Conclusion

The results of the present study showed that carvacrol not only has antibacterial properties but also can significantly enhance the efficacy of cefixime against planktonic form of *E. coli.*

Carvacrol also was found to have anti-biofilm activity. Additionally, carvacrol alone and also in combination with cefixime significantly decreased the expression of *luxS* and *pfs* genes compared to the control group. In light of these results, carvacrol can be introduced as an antibacterial and anti-virulence drug against *E. coli*. For therapeutic use, further studies are needed to confirm the safety of this compound.

## Material and methods

### Bacterial strain culture media and chemicals

*E. coli* ATCC 35,218 was used as the reference strain. This bacterium was obtained from the Persian Type Culture Collection (Iran). Inocula were prepared by 16 h aerobically culture in Mueller–Hinton Broth (MHB; Oxoid) at 37 °C. All bacterial culture media were obtained from Merck (Darmstadt, Germany). Carvacrol was acquired from Sigma-Aldrich (UK; 98%; CAS number 499–75-2). Cefixime was purchased from Merck (Darmstadt, Germany).

### Antibacterial activity

The antibacterial properties of carvacrol and cefixime were evaluated by the disk diffusion method and the determining Minimal inhibition concentration (MIC) and Minimal bactericidal concentration (MBC) values.

### Disk diffusion agar test

The disk diffusion method was performed to investigate the antibacterial properties of carvacrol and cefixime against *E. coli* according to CLSI (2019) guidelines and as reported by Humphries and co-workers [68]. The cefixime disk (5 µ) was prepared by Padtan Teb (Tehran, Iran). For carvacrol, sterile blank disks were inoculated with 20 µl of 250–1000 μg/mL of this agent. In detail, the inocula of bacteria were prepared to approximately 10^5^ CFU/mL with sterile saline solution. Using a sterile cotton swab Mueller–Hinton agar plates were cultured by this bacteria to get a uniform microbial growth on both control and test plates. Carvacrol.

was dissolved in 1% dimethylsulfoxide (DMSO) (for easy diffusion) and sterilized by a 0.45 μm membrane filter. Under aseptic conditions, empty sterilized discs (Whatman no. 5, 6 mm dia) were impregnated with 20 µLl of carvacrol solution and placed on the agar surface. A blank paper disc contained 20 µL of sterile saline solution plus 1% DNSO was used as a negative control. The plates were left for 30 min at room temperature to allow the diffusion of agent, and then they were incubated at 37 °C for 24 h. After the incubation period, the zone of inhibition was measured with a calliper. The results were analysed by t-test using SPSS software package version 13.0 for windows.

### The microdiluition broth assay

The MIC values were determined for both carvacrol and cefixime using a microdilution broth test according to CLSI (2019) as described elsewhere [69]. For carvacrol, two-fold serial dilutions of 1000 to 3.9 μg/mL were made in tryptic soy broth (TSB) plus 0.1% dimethyl sulfoxide (DMSO). DMSO was applied to increase solubility of carvacrol. Bacterial suspensions at (5 × 10^5^) CFU/mL then were added to all wells. The tube containing bacteria at (5 × 105) CFU/mL in sterile saline was considered to be the control. The microplates were incubated at 37 °C for 24 h. The MIC value of carvacrol was the lowest carvacrol concentration that produced no visible growth. The MIC determination test for cefixime was similar to that for carvacrol, except that the serial dilution was 1024–4.0 μg/mL. In order to investigate the MBC value, 10 μL from wells showing no visible growth in the microdilution broth test was inoculated onto tryptone soya agar (TSA). The MBC value was defined as the minimum concentration of the compound required to kill 99.9% of the bacteria [70]. These tests were performed in triplicate.

### Checkerboard method

The effect of the interaction between carvacrol and cefixime on *E. coli* was investigated using the checkerboard test on 96-well microtiter plates, as described elsewhere [71]. The results of this test were determined as the fractional inhibitory concentration index (FIC_i_). For testing, the microplate wells were arranged as follows: carvacrol was diluted two-fold along the x-axis and cefixime was diluted two-fold along the y-axis. The final volume in each well was 100 μL including 50 μL of carvacrol dilution and 50 μL of cefixime dilution. Subsequently, 100 μL of media containing 5 × 10^5^ CFU/mL of *E. coli* was added to each well. The plates then were incubated at 37 °C for 24 h. FIC_A_ was calculated as the MIC of A (carvacrol) in combination with cefixime/MIC of A alone. FIC_B_ was calculated as the MIC of B (cefixime) in combination with carvacrol/MIC of B alone. The index was calculated as FICi = FIC_A_ + FIC_B_. The results were interpreted as synergy (FICi ≤ 0.5), addition (0.5 < FICi ≤ 1), indifference (1 < FICi ≤ 2), and antagonism (FICi ˃ 2) [72, 73].

### Anti-biofilm activity

To investigate the anti-biofilm ability of the substances, the microtiter plate assay was applied. The sterile 96-well polystyrene microplates were filled with 80 μL of TSB plus 0.1% DMSO containing sub-MIC concentrations (MIC/2, MIC/4, and MIC/8) of carvacrol or cefixime (six wells for each concentration). Then, 20 μl of the inocula (~ 2 × 10^6^ CFU/mL) was added to each well (final concentration of bacteria was 5 × 10^5^ CFU/mL). The control contained TSB and 0.1% dimethyl sulfoxide with *E. coli*. The microplates were incubated without agitation at 37 °C for 24 h and the non-attached bacteria then were removed by three times washing of plates using phosphate buffered saline (PBS). The surface-attached cells were stained with crystal violet (0.1%) for 20 min. After that, the excess dye was removed and the microplates were washed with 300 μl PBS. The attached cells were solubilized by adding 100 μL of ethanol:acetic acid (95:5 v/v). Finally, the optical density (OD) of the wells was measured using a microplate reader (ELx808; BioTek; USA) at 560 nm. Each assay was done in triplicate and the data are presented as the mean ± SD (standard deviation). As a measure of efficacy, the anti-biofilm activity was determined as: percentage of inhibition = 100—[(OD of the treated wells)/(mean OD of negative control wells without antimicrobial agent) × 100)] [1].

### SEM of carvacrol and cefixime on structural cells and biofilm

The effect of carvacrol and cefixime alone and in combination on the cell structure and biofilm formation of *E. coli* was investigated using SEM. For this purpose, biofilm of *E. coli* ATCC 35,218 was prepared in 6-well microtiter plates. In detail, 1600 μL of TSB plus 0.1% DMSO containing MIC/2 concentration of carvacrol or cefixime were filled in 6-well polystyrene microplates. For combination condition TSB plus 0.1% DMSO containing MIC/2 concentrations of carvacrol and cefixime were added. Then, 400 μl of bacteria at ~ 2 × 106 CFU/mL was added to each well (final concentration of bacteria was 5 × 10^5^ CFU/mL). The control wells contained TSB plus 0.1% DMSO and bacteria and the treated biofilm groups contained medium with DMSO and bacteria and MIC/2 concentrations of carvacrol or cefixime alone or in combination. After an overnight of incubation at 37 °C, the samples were fixed in 2.5% buffered glutaraldehyde for 2 h at room temperature, followed by dehydration using ethanol concentrations of 50%, 70%, 80%, 90%, 95% and 98%. Each ethanol treatment lasted for 10 min at room temperature. The samples were stored at 4 °C for 1 h and then freeze-dried. Each sample was coated with gold and examined in a JEOL JSM-840 scanning electron microscope operating at an accelerating voltage of 15 kV.

### Effect of carvacrol and cefixime on QS-related gene expression

The effects of carvacrol and cefixime on the expression of the QS-related genes (*luxS* and *pfs*) of *E. coli* were examined using RT‐qPCR in the biofilm phase of growth. At first, the bacterial biofilm was developed as described in the presence of MIC/2 concentrations of carvacrol and cefixime. RNA then was extracted from the bacterial cells of the biofilm phase and, after conversion to cDNA, the main real-time PCR was performed.

### RNA extraction and cDNA synthesis

To obtain bacterial RNA, the cells were cultured in a 6-well microplate with MIC/2 of each agent (carvacrol or cefixime) and combination of carvacrol and cefixime (MIC/2 of carvacrol plus MIC/2 of cefixime). Three wells were considered as antimicrobial-free controls. After the incubation period at 37 °C, the non-adherent cells were removed and the well was washed with PBS. The adherent cells were scraped and processed for RNA extraction using a commercial RNA extraction and purification kit (Jena Bioscience; Germany) according to manufacturer instructions. The quality and quantity of the extracted RNA was checked by agarose gel electrophoresis and by using UV absorption at 260/280 nm. A commercial cDNA synthesis kit (SinaClon; Iran) was applied to obtain the cDNA. The synthesized cDNA was stored at -70 °C for further experiments.

### Real-time qRT-PCR reaction

The real-time process was performed using the SYBR Green master mix kit (Ampliqon; Denmark) and primers as described previously (Table 2). Quantitative gene expression of *luxS* and *pfs* was determined using real-time qRT-PCR according to the following cycle protocol: 4 min at 95 °C (denaturation) for 40 cycles, 15 s at 95 °C, 30 s at 56 °C, and 30 s at 72 °C. The *rrsD* gene was used as the reference gene. All the samples were analyzed in triplicate and, finally, relative gene expression analysis was performed using the 2^−ΔΔCT^ method [74].

**Table 2:** Primers used in present study [75]

Product size (bp)	Primer	
196	5′-ATACCGCATAACGTCGCAAG-3′	*rrsD*
	5′-ATATTCCCCACTGCTGCCTC-3′	
197	5′-AATCACCGTGTTCGATCTGC-3′	*luxS*
	5′- GCTCATCTGGCGTACCAATC-3′	
83	5′-ATCGTTGTCTCGGACGAAGC-3′	*pfs*
	5′-GGACAGCCTGGTAACTGACCG-3′	

### Statistical analysis

Data were analyzed using GraphPad Prism software (version 8). All experiments were performed in triplicate and one-way ANOVA was applied to analyze the differences among the treatments. In all cases, the level of significance was at least 0.05.

## Data Availability

The data presented in this study are available from the corresponding author upon request.

## Abbreviations

QS: Quorum Sensing
AI-2: Autoinducer 2
DMSO: Di Methyl Sulfoxide
CFU: Colony Forming Unite
